# Dr. John Frederic Haller

**Published:** 1911-01

**Authors:** 


Dr. John Frederic Haller, of Brooklyn, died of pneumonia at his home December 3, 1910, aged 48 years. He graduated at the Medical Department of the University, in 1888, served as a Medical Officer of Volunteers, during the war with Spain in 1898, and more recently was a physician to the Bushwick Hospital for Children. He had several other hospital services and was a member of the usual medical societies.

